# Percutaneous nephrolithotomy in horseshoe Kidneys: is rigid nephroscopy sufficient tool for complete clearance? a case series study

**DOI:** 10.1186/1471-2490-9-17

**Published:** 2009-11-16

**Authors:** Mohamed N El Ghoneimy, Ahmed S Kodera, Ashraf M Emran, Tamer Z Orban, Ahmed M Shaban, Mohamed M El Gammal

**Affiliations:** 1 Department of Urology, Cairo University hospitals, 1 Al-saray street, Al-Manial, Cairo 11559, Egypt

## Abstract

**Background:**

this study represents a case series to evaluate how successful is the rigid percutaneous nephroscopy as a tool for clearance of all stones in various locations in horseshoe kidneys.

**Methods:**

Between 2005 and 2009, we carried out PCNL (percutaneous nephrolithotomy) for calculi in horseshoe kidneys in 21 renal units (17 patients) in our department. The indications were large stone burden in 18 units and failed SWL(shock wave lithotripsy) in 3 renal units. All procedures were done under general anesthesia; using fluoroscopic guidance for localization and standard alkan dilatation followed by rigid nephroscopy and stone extraction with or without stone disintegration. We analyzed our results regarding the site and number of the required access, the intra and postoperative complications, the presence of any residual stones, as well as their location.

**Results:**

The procedure was completed, using a single access tract in 20 renal units, with the site of puncture being the upper calyx in nine units and the posterior middle calyx in eleven units. Only in one renal unit, two access tracts (an upper and a lower calyceal) were required for completion and a supracostal puncture was required in another case. There was no significant intraoperative bleeding and no blood transfusion was required in any patient. A pelvic perforation occurred in one case, requiring longer PCN (percutaneous nephrostomy) drainage. One patient with infection stones suffered urosepsis postoperatively which was successfully managed. Three cases had residual stones, all located in the renal isthmus, all residuals were un approachable with the rigid instrument; resulting in a overall stone-free rate of 85.7% at discharge.

**Conclusion:**

Percutaneous nephrolithotomy is generally safe and successful in the management of stones in horseshoe kidneys. However, location of the stones in these patients is crucial to decide the proper tool for optimal stone clearance result.

## Background

Horseshoe kidney occurs in about 1 in 400 persons [1]. As with other fusion anomalies, it is found more commonly in males. During embryogenesis, fusion of the lower poles prevents normal ascent and causes malrotation with anterior displacement of the collecting system. Insertion of the ureter on the renal pelvis is displaced superiorly and laterally, probably as the result of incomplete renal rotation. It is associated with a significant rate of ureteropelvic obstruction. These factors contribute to impaired drainage with stasis, infection and predispose to calculus formation. The incidence of stone formation in horseshoe kidneys has been reported to be approximately 20% [2].

Since the reports of Wickham and Kellet in 1981 and Clayman in 1983, percutaneous extraction of stones in horseshoe kidneys has been widely adopted as the standard of care for stones greater than 2 cm or when shock wave lithotripsy fails. While the atypical anatomical orientation of the calices and renal pelvis makes spontaneous passage of stones less likely; orientation of the calices and vessels renders percutaneous puncture of horseshoe kidney relatively safe[3].

Janetschek and Kunzel described three different arterial patterns supplying the horseshoe kidney: normal renal arteries, accessory arteries originating from different levels and entering renal hilum and aberrant arteries entering directly the poles or the isthmus of the kidney. Except for some of the arteries to the isthmus, there were no vessel on the dorsal aspect of the kidney; hence the risk of arterial bleeding is deemed not higher than with normal kidneys [4].

In horseshoe kidney, the frontal plane lies more or less in the sagittal plane of the body. Consequently, the posterior row of calices point dorsomedially and the ventral row dorsolaterally and the renal pelvis is in a ventral position. In some kidneys there are also calices to the isthmus. These always lie within a coronal plane and point medially [4] Generally, the orientation of the collecting system offers surprisingly good access to percutaneous nephrolithotomy. The calices pointing dorsally are entered by direct puncture; where as access to the calices in the isthmus is gained across the pelvis. The anatomical situation results in a lower and medial position of nephrostomy tract, whose orientation is more or less dorsoventral.

In this study we tried to critically evaluate our stone clearance success rates using rigid nephroscope only, in urolithiasis in a horse shoe kidney candidate for percutaneous stone extractions.

## Methods

Cairo university hospitals ethical committee granted ethical approval for the study. Our centre is a national tertiary referral centre for urological patients. Between 2005 and 2009, out of our patients records for percutaneous nephrolithitomy we identified a total of 17 patients (21 renal units) underwent percutaneous renal surgery for renal stones in horseshoe kidneys. In eighteen renal unit had a stone burden more than 2.5 cm; as measured by the length of the maximum diameter and were offered PCNL as a primary management. Three patients with stones less than 2 cm undertook PCNL, following failure of fragmentation after 3 sessions of SWL.

**Figure 1:**
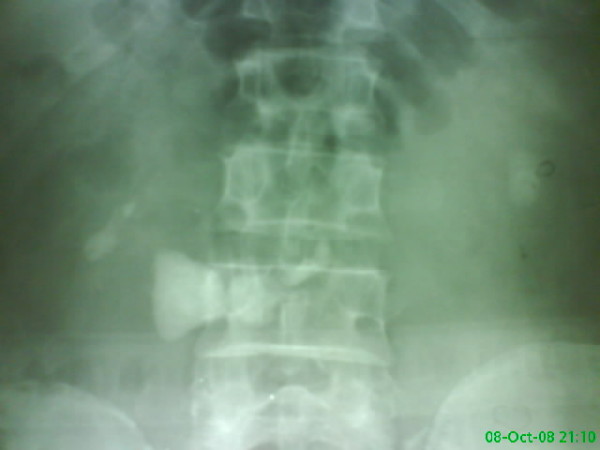
**KUB showing stones in a horseshoe kidney with stones extending in the isthmus**.

**Figure 2:**
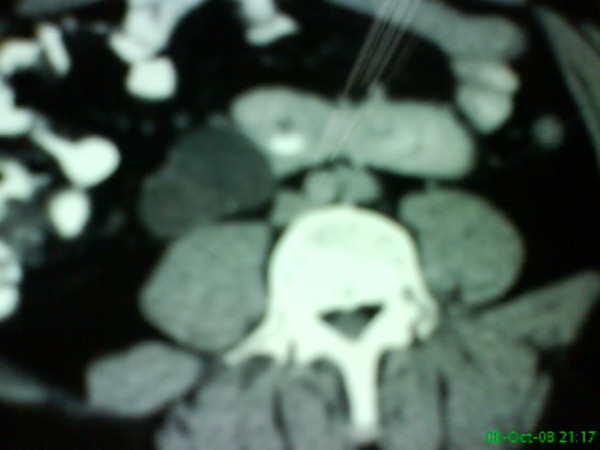
**CT scan showing isthmic stone in a horseshoe kidney**.

All patients were evaluated clinically and underwent routine laboratory investigations. Patients with positive urine cultures were started on an appropriate antibiotic. KUB and IVU were required for planning of the percutaneous access (Figures 1, 2) and US was done to assess the degree of hydronephrosis and the parenchymal thickness. CT angiography was performed only in the first 10 patients to delineate the vascular anatomy and its relation to the percutaneous access in all renal units, all vessels were found to enter from the medial aspect of the kidney and apart for some of the arteries to the isthmus, there were no vessels on the dorsal surface of the kidney.

### The procedure

After obtaining the patients' informed consent to carry out the surgery including their approval for potential use their anonyms medical data from our data base for research and audit purposes. a dose of perioperitave antibiotic is administered with the induction of general anesthesia. Cystoscopy and ureteric catheterisation are initially performed in the lithotomy position. In case of impacted stone in the pelvis or pelviureteric junction, an opened tipped 6F ureteric catheter is passed over a J-tip guide wire. The patient is then turned to the prone position. Bolsters are placed underneath the patient's abdomen in order to fix the kidney, pushing it posteriorly and limiting its movement during respiration.

With the C-arm in the vertical position, the pelvicalyceal system is opacified and distended with contrast material through the ureteric catheter. An 18-Gauge puncture needle is advanced in a straight line towards the desired calyx. The puncture site is always medial to posterior axillary line, with an angle of 70-90 degrees with the horizontal plane directed towards the targeted calyx. Due to the downward and medial displacement of the calyces, examination with C-arm at 90 degrees provides a direct end-on view of the posterior calyx. The C-arm is then rotated 30 degrees towards the surgeon & the depth of needle penetration is monitored fluoroscopically. The site of the puncture depends on the location and number of the stones as well as the orientation of the pelvicalyceal system. Whenever possible, the middle followed by the upper calyx are chosen to access to the collecting system. In patients with normal renal anatomy. Due to downward displacement of horseshoe kidneys, upper polar accesses are usually achieved through an infracostal puncture which is relatively safe away from the pleura.

**Figure 3:**
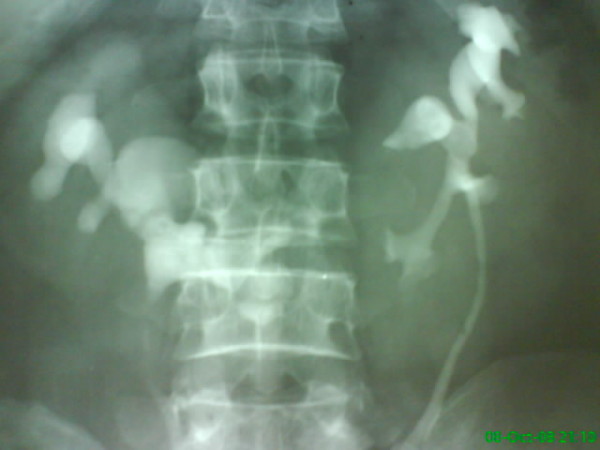
**preoperative IVU**.

Once the puncture was made, a guide wire is then passed into the collecting system, followed by dilation of the tract using the standard telescopic metal Alken set over a central rod. A 30 Fr. Amplatz sheath is then inserted (Figure 3). Because the tract is almost vertical, oblique fluoroscopy is needed to guide tract dilatation and placement of an operating sheath.

Nephroscopy is then carried out, using a rigid nephroscope. Following stone visualization, fragmentation and extraction were completed with the use of the Pneumatic Swiss lithoclast and stone grasper forceps. Because of the relatively long almost perpendicular distance from the skin to the renal pelvis in horseshoe kidneys, a middle calyceal puncture is usually preferred in cases with multiple stones, especially if lower calyceal or isthmic. An additional lower calyceal puncture was only required in one case with lower calyceal and isthmic stones, where it allowed for retrieval of the lower calyceal stones by compensating for the distance factor, but it failed to allow us retrieving the isthmic stones as the rigidity and length of the tract does not permit maneuverability of the rigid nephroscope inside the collecting system. The procedure is left tubeless, whenever possible.

A KUB is performed 24 hours later to detect any missed stones and an antegrade or retrograde study is carried out prior to removal of the ureteric catheter or the nephrostomy tube to exclude significant extravasation. Complications and stone-free rates were recorded. Complications were characterized as major if they required additional intervention or resulted in a prolonged hospital stay or minor if they could be managed conservatively with no additional intervention or morbidity.

## Results

Patients' age ranged between 21-51 years with a mean age of 34 years. Overall, of the 17 patients, there were 13 males and 4 females (21 renal units). Two patients had history of prior renal surgery (pyelolithotomy in one and hemi-nephrectomy on the opposite side in another case). Three patients had tried SWL with failure of fragmentation after 3 sessions. All patients had normal kidney functions and none suffered from any bleeding disorder. Eleven renal units (52.4%) had only pelvic stones, 6 units (28.6%) had pelvic and lower calyceal stones, three cases (14.2%) had stones in the pelvis, lower calyx and isthmus and in one case (4.8%) stones were upper calyceal only. The stone size was calculated by measuring the length of the maximum stone diameter. In cases of multiple stones, the length of the maximum diameter of each stone was added to calculate the size. Eleven renal units (52.4%) had stone sizes from 2-6 cm, 10 units (47.6%) had stones > 6 cm.

The procedure was carried out through a single access tract in 20 out of the 21 endoscopies (95.2%). However, two access tracts were needed in 1 renal unit (4.8%). In 9 units (42.85%), the upper calyx alone was the site of puncture. Posterior middle calyx alone was chosen in 11 cases (52.4%). Two punctures were used for completion of the procedure in 1 case (4.8%) and the combination was an upper and lower calyceal approach. Supracostal puncture was done in one case (4.8%). The operative time ranged from 30 min to 160 min (mean 70 min).

*Intra-operative bleeding *was not significant; none of the cases required blood transfusion intra or postoperatively. *Calyceal neck injury *occurred in one case, causing minor bleeding that slightly impaired the vision, but the procedure was completed with successful stone clearance. A major pelvic *perforation *occurred in one case during tract dilatation and was managed conservatively with prolonged drainage with a nephrostomy tube. Antegrade pyelography on day 5 revealed no extravasation.

**Figure 4:**
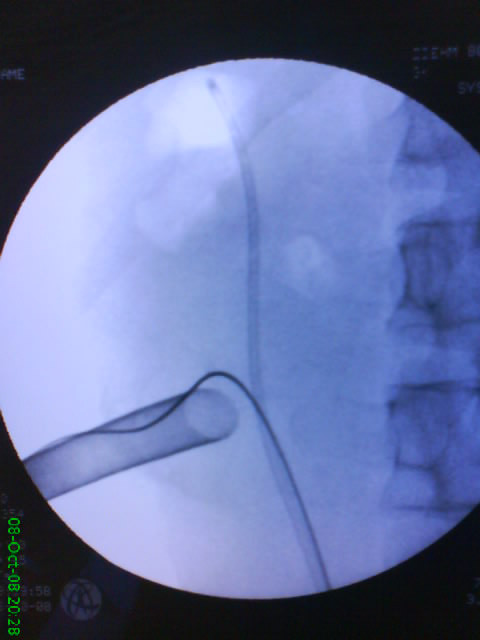
**intraoperative fluoroscopic image showing access to the lower pole**.

*Residual stones *were left in three cases (14.3%). In all cases, they were located in the renal isthmus (0.5-1.5 Cm). In two cases, they were recognized intra-operatively with fluoroscopy and in the third, in the postoperative KUB as it lied over the shadow of the spine. They were all inaccessible from the calyx of entry, as the long nephroscope barely reached the renal isthmus and the perpendicular access did not allow manipulation inside the collecting system. We believe a flexible nephroscope would have achieved complete successful clearance in the two cases recognized intra-operatively; however, unfortunately it was not available in our institute at that time period. A direct isthmic puncture was deemed too hazardous. These three cases were offered post-operative SWL vs conservative management and they all chose to be placed under follow-up (Figure 4).

Two cases among the 21 renal units (9.5%) suffered self-limited leakage from the nephroscopy site for 12-24 hours. None of them required secondary intervention. Post-operative urosepsis occurred in a young female with bilateral infection stones. In spite of pre-operative antibiotics, she developed post-operative persistent fever and leucocytosis with profound hypotension. After exclusion of any perirenal collection related to the procedure She was referred to the ICU and responded to hemodynamic support and precise antimicrobial therapy. No bowel injury was encountered in our study; a fact that we attributed to the very medial location of the percutaneous tract, in comparison to standard PCNL; in which bowel injury is also considered uncommon. None of the cases included in our study required secondary intervention.

## Discussion

Various treatment modalities have been used to treat stones in horseshoe kidneys, including shock wave lithotripsy (SWL), ureteroscopy, PCNL, and open surgery. Although adequate fragmentation can be achieved by SWL, yet the anatomic abnormalities may prevent fragment passage in a substantial number of patients. The overall stone-free rate has been only around 53% (range 50% to 79%)[2]. Alternatively, PCNL has been used successfully to remove calculi from horseshoe kidneys. The upper pole and mid renal, but not the lower pole calyces, which are located posteriorly, are recommended. The inferior lie of the kidneys places most upper pole calyces below the twelfth rib, thereby making a supracostal puncture a relatively safe access.

The use of PCNL in the treatment of stones in horseshoe kidneys has received little attention in published series. Most reported on a small number of patients. The largest series in English published studies included 47 patients with 60 renal units treated collected from data over a 17 years period by Stephanie et al[5]. Another study by Mansoura University presented their experience on 34 patients with 45 stone-bearing horseshoe kidneys treated by PCNL in a period of 8 years [6]. table 1 shows the results of percutaneous nephrolithotomy in horseshoe kidneys in different studies.

**Table 1:** Results of different studies done on percutaneous nephrolithotomy in horseshoe kidney

References	No. of patients	% upper Pole access	%complications (minor/major)	%initial Stone free rate	%secondary procedures
Jones et al [7]	15	Not available	26(20/6)	72.3	13.3

Al-Otaibi and Hosking [8]	12	75	42(42/0)	75	8.3

Lingerman and Saw [9]	17	81	29(24/5)	84.6	73

Shokeir et al [6]	34	46	13(0/13)	82	35.3%

Stephanie et al [5]	47	48	23(9/2)	88	34%

Present study	17	48	19(14/5)	85.7	0

The percentage of upper pole access in previous studies ranged from 62% to 81%. This is because it allows access to the upper pole calices, renal pelvis, lower pole calices, pelviureteric junction and proximal ureter. Furthermore, it can decrease blood loss because the long axis of nephroscope is aligned with the long axis of the kidney, thereby minimizing nephroscope torque on renal tissue during manipulation. Unfortunately, upper pole access results in an unusually long tract, and the instruments may not reach the lower and medial calyces. In our work, the access to the kidney was through upper calyx in 42.8% of cases; whereas a middle calyceal puncture was resorted to in 52.4% of cases. This can be attributed to the fact that 42.8% of our renal units had lower calyceal stones. A middle calyceal access was expected to shorten the distance to the lower calyx substantially.

Three of our patients had residual isthmic stones; that were inaccessible using the rigid nephroscope. We believe flexible nephroscopy to be a vital part of rendering patients stone-free during PCNL in anomalous horseshoe kidneys, especially if associated with isthmic stones. Even though flexible nephroscope was not available in our unit, our stone-free rate (85.7%) is still comparable to other working groups with rates ranging from 72% to 87.5%.

## Conclusion

Percutaneous nephrolithotomy is a safe and effective method in treating stones in horseshoe kidney. The procedure offers the highest likelihood of rendering patients stone free. However, patients with stones in an isthmic location the availability of flexible nephroscope might achieve a better clearance.

## Competing interests

The authors declare that they have no competing interests.

## Authors' contributions

AK: data collection and querying the hospital records. AS: data collection & participation in writing the manuscript. ME: design the study &writing the manuscript. AE: drafting the manuscript. TO: participation in the design of the study &interpretation of the results. ME: general supervision. All authors read and approved the final manuscript

## Pre-publication history

The pre-publication history for this paper can be accessed here:

http://www.biomedcentral.com/1471-2490/9/17/prepub
